# A novel *NFKB1* variant in a Japanese pedigree with common variable immunodeficiency

**DOI:** 10.1038/s41439-024-00271-2

**Published:** 2024-03-22

**Authors:** Naoko Nakatani, Akihiro Tamura, Hiroaki Hanafusa, Nanako Nino, Nobuyuki Yamamoto, Hiroyuki Awano, Yasuhiro Tanaka, Naoya Morisada, Suguru Uemura, Atsuro Saito, Daiichiro Hasegawa, Kandai Nozu, Yoshiyuki Kosaka

**Affiliations:** 1 Department of Pediatrics, Kobe University Graduate School of Medicine, Kobe, Japan; 2 Department of Pediatrics, Hyogo Prefectural Harima-Himeji General Medical Center, Himeji, Japan; 3 Research Initiative Center, Organization for Research Initiative and Promotion, Tottori University, Tottori, Japan; 4 Department of Hematology, Shinko Hospital, Kobe, Japan; 5 Department of Clinical Genetics, Hyogo Prefectural Kobe Children’s Hospital, Kobe, Japan; 6 Department of Hematology and Oncology, Hyogo Prefectural Kobe Children’s Hospital, Kobe, Japan

**Keywords:** Immunological deficiency syndromes, Clinical epigenetics

## Abstract

Recently, heterozygous loss-of-function *NFKB1* variants were identified as the primary cause of common variable immunodeficiency (CVID) in the European population. However, pathogenic *NFKB1* variants have never been reported in the Japanese population. We present a 29-year-old Japanese woman with CVID. A novel variant, c.136 C > T, p.(Gln46*), was identified in *NFKB1*. Her mother and daughter carried the same variant, demonstrating the first Japanese pedigree with an *NFKB1* pathogenic variant.

The most common primary immunodeficiency disease characterized by defective antibody production is common variable immunodeficiency (CVID)^1^. CVID is a clinically and genetically heterogeneous disorder characterized by impaired antibody production and recurrent infections. However, the genetic cause of CVID in the majority of patients remains unknown. Recent large-scale whole-genome sequencing analysis identified *NFKB1* as the most common causative gene of CVID, accounting for 4% of CVID cases in a predominantly European population^2^. No pathogenic variants in *NFKB1* have been reported in the Japanese population to date.

The proband was a 29-year-old female patient whose medical history consisted only of an annual fever and rash. She exhibited mild flexion restriction in the right middle finger and left ring finger at the proximal interphalangeal joint since her late teens, with occasional pain in the wrist and ankle. Her 54-year-old mother had rheumatoid arthritis since the age of 52 and was prescribed methotrexate. Her 57-year-old father, two brothers aged 32 and 25 years, and three-year-old daughter were healthy (Fig. 1A). When she visited a dermatologist for two weeks of fever and stomatitis, she was found to have marked hypogammaglobulinemia, with immunoglobulin G (IgG), IgG2, IgA, and IgM levels less than 0.06, 0, 0, and less than 0.02 g/L, respectively. The absolute lymphocyte count was 1740 cells/μl, while the white blood cell count was 7900 cells/μl. The percentage of CD19^+^ B cells within the total lymphocyte population was markedly reduced to 0.2%. According to flow cytometric analyses, the counts of other peripheral blood lymphocyte subsets were within the normal ranges as follows: CD3^+^ T cells, 92%; CD4^+^ T cells, 50%; CD8^+^ T cells, 36%; and CD16^+^ CD56^+^ natural killer (NK) cells, 7.6%.

**Fig. 1:**
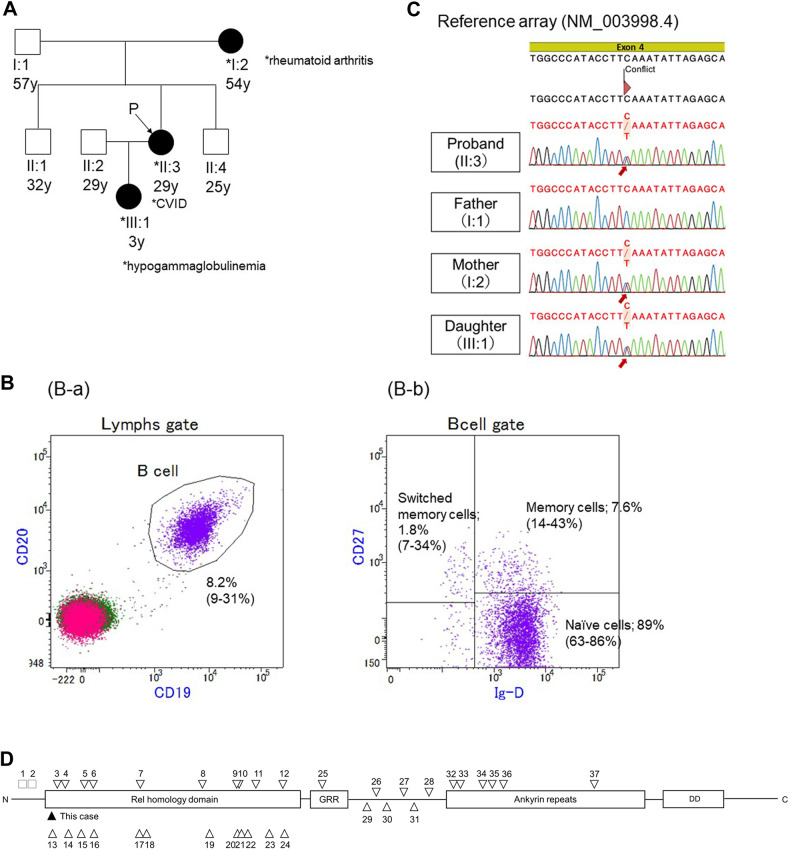
Patient details and *NFKB1* variants previously reported. **A** Pedigree of the family. The proband’s 54-year-old mother (I:2) suffered from rheumatoid arthritis. Her 57-year-old father (I:1), two brothers aged 32 (II:1) and 25 years (II:4), and a 3-year-old daughter (III:1) were healthy. **B** Flow cytometric analysis of B-cell subsets in the peripheral blood of the proband’s daughter. (B-a) Flow cytometric analysis of CD19^+^ CD20^+^ B cells among total lymphocytes. (B-b) Flow cytometric analysis of IgD^+^ CD27^−^ naive B cells, IgD^+^ CD27^+^ IgM memory B cells, and IgD^−^ CD27^+^ switched B cells within the subset of CD19^+^ CD20^+^ B cells. **C** Electropherogram of Sanger sequencing revealing the same variant, c.136 C > T (p.Gln46*), in *NFKB1* in the proband, mother, and daughter. **D** Schematic representation of the protein domains and previously reported genetic variants in *NFKB1*. The black and white arrowheads indicate this patient and previously reported patients, respectively. RHD Rel homology domain, GRR glycine-rich region, DD death domain.

The results of ^3^H-thymidine incorporation assays induced by phytohemagglutinin and concanavalin A were normal at 76,100 cpm (stimulation index: 127–456) and 69,700 cpm (stimulation index: 127–456), respectively. A Cr^51^ release assay was used to assess NK cytotoxicity, and the result of 22% was normal. Antibodies specific for Japanese encephalitis, measles, and rubella were all negative although the patient had been vaccinated against these antigens. Abdominal ultrasonography revealed no hepatosplenomegaly or intra-abdominal lymph node enlargement. Chest computed tomography revealed no thymoma suggestive of Good’s syndrome, and chronic airway inflammation due to repeated infections was not observed.

The patient was diagnosed with CVID based on a markedly reduced serum IgG level accompanied by decreased IgA and IgM, an absent antibody response to vaccinations, and the lack of other causes of immunodeficiency. The proband and her parents provided written informed consent for genetic testing following genetic counseling. The Medical Ethics Committee of Kobe University, Kobe, Japan (IRB number: B210180) approved this genetic study. Peripheral blood leukocytes were isolated from the proband and her parents. The QuickGene-Auto 12 S system (Wako Pure Chemical Industries, Ltd., Tokyo, Japan) was used to extract genomic DNA following the manufacturer’s instructions. An Illumina NextSeq 2000 (Illumina, San Diego, CA) and Twist Comprehensive Exome Panel (TWIST Bioscience, South San Francisco, CA) were used for whole-exome sequencing (WES).

A novel heterozygous nonsense variant of *NFKB1* [NM_003998.4: c.136 C > T, p.(Gln46*)] was identified in the region encoding the N-terminal Rel homology domain (RHD) by WES; Sanger sequencing was subsequently performed in the proband and her mother (Fig. 1C). This variant was not reported in the Human Gene Mutation Database (https://www.hgvd.genome.med.kyoto-u.ac.jp/). The variant was classified as pathogenic (pathogenic very strong (PVS) 1 + pathogenic moderate (PM) 2 + pathogenic supporting (PP) 1) according to the American College of Medical Genetics and Genomics guidelines because the loss of *NFKB1* function is known to cause CVID, and the variant identified in the proband and her mother was null.

Although the proband’s daughter was asymptomatic, she underwent an immunological examination because of the autosomal dominant inheritance pattern of *NFKB1*. The daughter had reduced IgG, IgG2, IgM, and IgA levels of 3.0 g/L, 0.41 g/L, 0.22 g/L, and 0.04 g/L, respectively. These values were more than 2 SDs below the age-specific normal ranges^3^. The absolute lymphocyte count was 5100 cells/μl, while the white blood cell count was 8300 cells/μl. The percentage of CD19^+^ B cells within the total lymphocyte population was 8.2%, and the absolute count was 420 cells/μl. The median percentage and absolute count of CD19^+^ B cells in 3-year-old children are 17% (10th–90th percentile: 9–31%) and 590 cells/μl (10th–90th percentile: 310–1130 cells/μl)^4^, respectively. Therefore, the daughter’s B-cell counts were not significantly lower than the age-specific normal values. The percentages (median and 10th–90th percentile of 3-year-old children^4^) of IgD^+^ CD27^−^ naive cells, IgD^+^ CD27^+^ memory cells, and IgD^−^ CD27^+^ switched memory cells among total B cells were 89.0% (74% and 63–86%), 7.6% (22% and 14–43%), and 1.8% (17% and 7–34%), respectively (Fig. 1B). These results indicated that impaired development from naive B cells to switched memory B cells was the cause of defective antibody production. Similarly, a reduced number of memory B cells in patients with *NFKB1* haploinsufficiency has been reported^2,5–7^. Intriguingly, the IgG levels and percentage of CD19^+^ B cells within the total lymphocyte population in the proband’s mother were 8.2 g/dl and 11%, respectively, which were within the normal range despite the fact that she carried the same *NFKB1* pathogenic variant. The daughter had hypogammaglobulinemia; thus, genetic testing was performed after obtaining written informed consent from her parents. The results revealed the same *NFKB1* variant in the daughter (Fig. 1C).

Immunoglobulin replacement therapy for severe hypogammaglobulinemia and recurrent infections was initiated in the proband. Her fever resolved after initiating immunoglobulin replacement therapy.

Observation was chosen for the proband’s mother, who did not have overt hypogammaglobulinemia, and the proband’s daughter, who had hypogammaglobulinemia but was asymptomatic.

Here, we report a Japanese family with CVID harboring a novel c.136 C > T variant in *NFKB1*. To our knowledge, this is the first reported case of a pathogenic *NFKB1* variant in a Japanese patient with CVID.

Nuclear factor kappa B (NFκB) is a transcription factor consisting of RelA (p65), RelB, C-Rel, NFκB1 (precursor p105/activator p50), and NFκB2 (precursor p100/activator p52). NFκB regulates more than 500 target genes and is involved in various signal transduction pathways, such as cell differentiation, survival, inflammatory response, and immunosuppression^8^. Loss of NFκB function is associated with CVID development, which is characterized by susceptibility to infection due to impaired development of antibody-producing mature B cells. The typical age for CVID diagnosis is 20–40 years^2^. Serum IgG levels and absolute B-cell numbers are variable^2^. Hypogammaglobulinemia is the most common manifestation of *NFKB1* haploinsufficiency, followed by respiratory tract infections and abscess formation^6^. Autoimmune diseases have been reported^6^, which is consistent with the mother’s rheumatoid arthritis. Approximately 15% of patients develop malignancies^6^; thus, careful follow-up is needed. Other less common symptoms include gut involvement, hepatosplenomegaly, lymphadenopathy, recurrent or chronic diarrhea, and Epstein–Barr virus infection^6^.

Recent large-scale whole-genome sequencing analysis identified pathogenic *NFKB1* variants in 16 of 390 (4%) patients with CVID in a European cohort^2^. Some genetic abnormalities involving *NFKB2*^9–11^ have been identified in Japanese CVID patients^12^, although *NFKB1* pathogenic variants have never been reported.

Figure 1D and Table 1 summarize previously reported *NFKB1* pathogenic variants. *NFKB1* consists of 24 exons (GenBank annotation NC_000004.11: 103422486-103538459) and encodes the 969 amino acid p105 and its shorter isoform 2 (968 amino acids). In order from the N-terminus, its structure consists of a Rel homology domain (RHD), a glycine-rich region, an ankyrin repeat, and a death domain (DD). The RHD mediates dimerization, specific protein inhibitor interactions, and DNA binding^13^. Most of the previously reported pathogenic variants were localized in the RHD (Fig. 1D). The variant identified in this patient was located on the N-terminal side among the previously reported variants, except for two large deletions (Fig. 1D).

**Table 1:** Summary of previously reported *NFKB1*variants.

	cDNA	Protein change	Familial/Sporadic	Age at diagnosis	IgG(g/L)	Reference
This case	c.136 C > T	Gln46*	Familial	29	<0.06	-
1	del 103370996-103528207		Sporadic	18	0.03	^2^
2	del 103436974-103652655		Sporadic	14	<0.1	^2^
3	c.137del	Ile47Tyrfs*2	Sporadic	14	4.85	^15^
4	c.160-1 G > A	NA	Familial	77	NA	^2^
5	c.187del	Glu63Lysfs*64	Familial	47	NA	^2^
6	c.200 A > G	His67Arg	Familial	44	NA	^14^
7	c.259-4 A > G	intronic	Sporadic	21	NA	^16^
8	c.260 T > G	Ile87Ser	Sporadic	NA	NA	^2^
9	c.293 T > A	Val98Asp	Sporadic	NA	NA	^2^
10	c.295 C > T	Gln99*	Sporadic	20	NA	^2^
11	c.465dupA	Ala156Serfs*12	Familial	2	5.17	^13^
12	c.469 C > T	Arg157*	Familial	48	NA	^17^
13	c.470 G > C	Arg157Pro	Familial	42	<0.33	^5^
14	c.494delG	Gly165Afs*32	Familial	NA	2.32	^18^
15	c.705 G > A	NA	Familial	3	9.58	^8^
16	c.730+4 A > G	ASsp191_Lys244delinsGlu	Sporadic	7	1.6	^19^
17	c.778_779insCTGTC	Gly261Valfs*5	Familial	44	NA	^14^
18	c.830dup	Lys278Glufs*3	Sporadic	NA	NA	^2^
19	c.835+2 T > G	Lys244_Asp279delinsAsn	Familial	64	1.42	^13^
20	c.843 C > G	Ile281Met	Sporadic	NA	NA	^2^
21	c.850 C > T	Arg284*	Familial	21	NA	^2^
22	c.874delG	Gly292Valfs*140	Familial	16	2.11	^5^
23	c.904dupT	Ser302Phefs*7	Familial	35	4.38	^5^
24	c.957 T > A	Tyr319*	Sporadic	19	NA	^16^
25	c.1005del	Arg336Glyfs*96	Sporadic	NA	NA	^2^
26	c.1012delT	Ser338Leufs*94	Familial	52	6.05	^5^
27	c.1149delT	His352Arg	Familial	34	1.03	^6^
28	c.1365delT	Val456*	Sporadic	43	0.08	^20^
29	c.1375delT	Phe459Leufs*26	Sporadic	7	NA	^16^
30	c.1423del	Ala475Profs*10	Familial	47	NA	^2^
31	c.1301-1 G > A	intronic	Sporadic	19	<0.51	^16^
32	c.1517delC	Ala506Valfs*17	Sporadic	13	1.6	^20^
33	c.1539_1543del	His513Glnfs*28	Familial	45	NA	^2^
34	c.1621_1622del	Asp541*	Familial	34	NA	^2^
35	c.1659C>G	Ile553Met	Familial	36	NA	^7^
36	c.1726dupA	Ile567Asnfs*6	Sporadic	42	2.14	^5^
37	c.2041 C > T	Gln681*	Familial	10	NA	^14^

*NFKB1* exhibits an autosomal dominant inheritance pattern, but differences in symptoms among relatives with the same *NFKB1* variant have been reported^6,13,14^.

The characteristics of the cohort of *NFKB1* variant carriers indicate incomplete clinical penetrance even with advancing age. This is likely due to the involvement of other genetic, epigenetic, or environmental factors in CVID development. Individualized follow-up for each patient is required because of the variation in immunological and other manifestations even between individuals within the same pedigree harboring the same *NFKB1* pathogenic variant. Additionally, our immunological study of the proband’s daughter provides valuable information regarding the early steps in the development of overt clinical disease.

We reported the first Japanese patient with the pathogenic *NFKB1* variant [c.136 C > T, p.(Gln46*)]. Clinical manifestations are highly variable even among family members having the same *NFKB1* variant. Genetic testing can be useful for managing patients with CVID.

## Data Availability

The relevant data from this Data Report are hosted at the Human Genome Variation Database at 10.6084/m9.figshare.hgv.3379.
